# Cyclosporin A Treatment of *Leishmania donovani* Reveals Stage-Specific Functions of Cyclophilins in Parasite Proliferation and Viability

**DOI:** 10.1371/journal.pntd.0000729

**Published:** 2010-06-29

**Authors:** Wai-Lok Yau, Thierry Blisnick, Jean-François Taly, Manuela Helmer-Citterich, Cordelia Schiene-Fischer, Olivier Leclercq, Jing Li, Dirk Schmidt-Arras, Miguel A. Morales, Cedric Notredame, Daniel Romo, Philippe Bastin, Gerald F. Späth

**Affiliations:** 1 G5 Virulence Parasitaire, Institut Pasteur and CNRS URA 2581, Paris, France; 2 Trypanosome Cell Biology Unit, Institut Pasteur and CNRS URA 2581, Paris, France; 3 Centre de Regulacio Genomica (CRG), Universitat Pompeus Fabre, Barcelona, Spain; 4 Centre for Molecular Bioinformatics, Department of Biology, University of Rome Tor Vergata, Rome, Italy; 5 Max-Planck Research Unit for Enzymology of Protein Folding, Halle/Saale, Germany; 6 Texas A&M Natural Products LINCHPIN Laboratory, Department of Chemistry, Texas A&M University, College Station, Texas, United States of America; 7 Department of Chemistry, Texas A&M University, College Station, Texas, United States of America; Louisiana State University, United States of America

## Abstract

**Background:**

Cyclosporin A (CsA) has important anti-microbial activity against parasites of the genus *Leishmania*, suggesting CsA-binding cyclophilins (CyPs) as potential drug targets. However, no information is available on the genetic diversity of this important protein family, and the mechanisms underlying the cytotoxic effects of CsA on intracellular amastigotes are only poorly understood. Here, we performed a first genome-wide analysis of *Leishmania* CyPs and investigated the effects of CsA on host-free *L. donovani* amastigotes in order to elucidate the relevance of these parasite proteins for drug development.

**Methodology/Principal Findings:**

Multiple sequence alignment and cluster analysis identified 17 *Leishmania* CyPs with significant sequence differences to human CyPs, but with highly conserved functional residues implicated in PPIase function and CsA binding. CsA treatment of promastigotes resulted in a dose-dependent inhibition of cell growth with an IC50 between 15 and 20 µM as demonstrated by proliferation assay and cell cycle analysis. Scanning electron microscopy revealed striking morphological changes in CsA treated promastigotes reminiscent to developing amastigotes, suggesting a role for parasite CyPs in *Leishmania* differentiation. In contrast to promastigotes, CsA was highly toxic to amastigotes with an IC50 between 5 and 10 µM, revealing for the first time a direct lethal effect of CsA on the pathogenic mammalian stage linked to parasite thermotolerance, independent from host CyPs. Structural modeling, enrichment of CsA-binding proteins from parasite extracts by FPLC, and PPIase activity assays revealed direct interaction of the inhibitor with LmaCyP40, a bifunctional cyclophilin with potential co-chaperone function.

**Conclusions/Significance:**

The evolutionary expansion of the *Leishmania* CyP protein family and the toxicity of CsA on host-free amastigotes suggest important roles of PPIases in parasite biology and implicate *Leishmania* CyPs in key processes relevant for parasite proliferation and viability. The requirement of *Leishmania* CyP functions for intracellular parasite survival and their substantial divergence form host CyPs defines these proteins as prime drug targets.

## Author Summary

Visceral leishmanisasis, also known as Kala Azar, is caused by the protozoan parasite *Leishmania donovani*. The *L. donovani* infectious cycle comprises two developmental stages, a motile promastigote stage that proliferates inside the digestive tract of the phlebotomine insect host, and a non-motile amastigote stage that differentiates inside the macrophages of mammalian hosts. Intracellular parasite survival in mouse and macrophage infection assays has been shown to be strongly compromised in the presence of the inhibitor cyclosporin A (CsA), which binds to members of the cyclophilin (CyP) protein family. It has been suggested that the toxic effects of CsA on amastigotes occurs indirectly via host cyclophilins, which may be required for intracellular parasite development and growth. Using a host-free *L. donovani* culture system we revealed for the first time a direct and stage-specific effect of CsA on promastigote growth and amastigote viability. We provided evidence that parasite killing occurs through a heat sensitivity mechanism likely due to direct inhibition of the co-chaperone cyclophilin 40. Our data allow important new insights into the function of the *Leishmania* CyP protein family in differentiation, growth, and intracellular survival, and define this class of molecules as important drug targets.

## Introduction

The cyclophilin (CyP) protein family consists of highly conserved proteins that share a common signature region of approximately 109 amino acids, the cyclophilin-like domain (CLD, Prosite access number: PS50072). The CLD carries the peptidylprolyl isomerase (PPIase) activity characteristic of CyPs [1], which has been implicated in protein folding, assembly of multi-protein complexes, and signal transduction [2]–[4]. CyPs are characterized by the binding of the cyclic peptide inhibitor cyclosporin A (CsA), which inhibits the protein phosphatase calcineurin and finds application for example as immune-suppressive drug in organ transplantation [5]. In addition to its inhibitory effect on T cell-mediated immunity [6]–[8], CsA displays anti-microbial activity against a variety of protozoan pathogens [9]–[11], including *Leishmania* [12]–[15].

Parasites of the genus *Leishmania* cause important human diseases collectively termed leishmaniasis, which range from mild, self-healing cutaneous lesions generated by *L. major* to fatal visceral infection of liver and spleen caused by *L. donovani* [16], [17]. *Leishmania* is transmitted by infected sand flies, which harbor the proliferating flagellate promastigote form of the parasite. Highly infectious metacyclic promastigotes are inoculated into the mammalian host during sand fly blood feeding, where they are engulfed by phagocytes of the endo-reticular system and develop inside the phagolysosome into amastigotes, which subvert the host immune response and cause the immunopathologies characteristic of the various forms of leishmaniasis [18], [19].

CsA has been shown to exert a leishmanicidal effect on intracellular *L. tropica* [12] and *L. major* in mouse and macrophage infection [13]–[15]. Although these findings define members of the *Leishmania* CyP protein family as potential important drug targets, only little is known on this protein family in trypanosomatids and the mechanisms of the anti-parasitic effects of CsA on intracellular *Leishmania* remain elusive. A potential role of *Leishmania* CyPs in amastigote differentiation and virulence can be postulated based on the role of *Leishmania donovani* LdCyP in disaggregation of adenosine kinase aggregates [20], an important enzyme in the *Leishmania* purine salvage pathway, whose activity substantially increases during the pro- to amastigote differentiation [21]. Furthermore, the amastigote-specific phosphorylation of cyclophilin 40 [22], [23] suggests that activity, localization, and interaction of this protein may be regulated in a stage-specific manner by post-translational modification.

The use of CsA for anti-leishmanial chemotherapy is limited by its suppressive action on host immunity, which leads to aggravation of experimental visceral leishmaniasis [24]. A better understanding on the biology of *Leishmania* CyPs, and their structural and functional differences to human CyPs is required to pave the way for the identification of new inhibitors with increased specificity for parasite CyPs. Here we initiated a first genome-wide analysis of the *Leishmania* CyP protein family and used the *L. donovani* axenic culture system [25], [26] to investigate the effects of CsA on promastigotes and amastigotes in culture. Our data indicate substantial evolutionary divergence between parasite and host CyPs, which may be exploitable for drug development. We provide evidence for stage-specific functions of *Leishmania* CyPs in regulation of promastigote cell shape and proliferation, and amastigote thermotolerance. We demonstrate for the first time a stage-specific and direct toxic effect of CsA on host-free amastigotes, validating *Leishmania* CyPs as drug targets.

## Materials and Methods

### Parasite culture

*Leishmania donovani* strain 1S2D (MHOM/SD/62/1S-CL2D) clone LdB [27] was maintained at 26°C, pH 7.4 in M199 medium supplemented with 10% FCS, 20 mM HEPES pH 6.9, 12 mM NaHCO_3_, 2 mM glutamine, 1× RPMI 1640 vitamin mix, 10 µg/ml folic acid, 100 µM adenine, 30 µM hemin, 8 µM biopterin, 100 U/ml penicillin and 100 µg/ml streptomycin. Axenic amastigotes were differentiated at 37°C with 5% CO_2_, in RPMI 1640 medium pH 5.5 supplemented with 20% FCS, 2 mM glutamine, 28 mM MES, 1× RPMI 1640 vitamin mix, 10 µg/ml folic acid, 100 µM adenine, 1× RPMI 1640 amino acid mix, 100 U/ml of penicillin and 100 µg/ml of streptomycin.

### Cyclosporin A and FK506 treatment

Both cyclosporin A (CsA) isolated from *Tolypocladium inflatum* (Calbiochem) and FK506 isolated from *Streptomyces tsukubaensis* (A.G. Scientific) were dissolved in absolute ethanol at a final concentration of 10 mM and the stock was stored at −20°C. Log-phase promastiogtes (2×10^6^/ml) were cultured in medium containing either solvent, CsA or FK506 at concentrations ranging from 5 to 25 µM and incubated at 26°C, pH 7.4 for 48 hours unless otherwise specified. Axenic amastigotes were differentiated at 37°C for 72 hours and were incubated at a density of 2×10^6^ parasites/ml at 37°C with 5% CO_2_, pH 5.5 for 48 hours in medium containing either solvent, CsA or FK506 unless otherwise specified.

### Determination of *Leishmania* growth and viability

The growth of solvent treated cells controls and CsA treated parasites was determined using a CASY cell counter (Schärfe System) or determined microscopically by cell counting using 2% glutaraldeyhde fixed cells. Cell proliferation was determined by CellTiter-Blue assay (Promega) according to the manufacturer's instructions. Briefly, 20 µl of CellTiter-Blue was added to the cells in 96-well plate and incubated at 37°C for 4 hours. Fluorescence was measured (exλ = 560 nm; emλ = 590 nm) using a spectrometer SP-2000 (Safas). Results were expressed in % of fluorescence intensity compared to solvent treated cells control. The tests were performed in quadruplicate.

### Bioinformatics analysis

The sequences of human and *Leishmania* cyclophilins were retrieved using the UniProt (www.uniprot.org) and GeneDB (www.genedb.org) databases, respectively, and conserved protein domains were identified by ScanProsite (www.expasy.ch/tools/scanprosite). In order to determine the level of conservation of CLD domains across human and trypanosomatid parasites, all putative CLD containing proteins of the sequenced genomes of *L. major, L. infantum, L. barsiliensis, T. brucei, and T. cruzi* [28]–[30] were retrieved from the TriTrypDB database (http://tritrypdb.org/tritrypdb/) using HUMAN_PPIA as an initial query for PSI-BLAST. After three cycles, all hits with a significant E-value (<10E-5) and more than 70% coverage of the CLD domain were selected, and their putative CLD domain was then extracted using the alignment to HUMAN_PPIA as a guide. Given the high level of conservation of the CLD domains, it is realistic to consider this dataset as a complete set of the CLD proteins present in the species covered by the current release of TriTrypDB (Release 1.1). These sequences were aligned with T-Coffee (default mode) [31], and a Neighbor-Joining tree was computed with 500 bootstrap replicates. Positions in contact between CsA and cyclophilin A were identified on the multiple sequence alignment and the corresponding columns were extracted. The resulting functional residues were compared and clustered for similarity using UPGMA.

### Structural modeling

We first identified *Leishmania* CyPs that are predicted to bind CsA using multiple sequence alignment of human and *Leishmania major* cyclophilins, and assessing the conservation of the residues known to be involved in cyclosporin A binding in known complexes. Based on these criteria, six *Leishmania major* cyclophilins shared the CsA binding residues with human PPIA or PPID (LmaCyP1, LmaCyP2, LmaCyP4, LmaCyP5, LmaCyP11 and LmaCyP40) and were selected for further analysis. The leishmanial cyclophilins were modelled with the automated mode of the Swiss-Model tool [32] using the following PDB structures as templates: 2 bit [33] for LmaCyp1; 3eov [34] for LmaCyP2, 2hqj (Arakaki and Merritt, unpublished), corresponding to LmaCyP11, for LmaCyP4 and LmaCyP5; 1ihg [35] for LmaCyP40. For each model or structure, the corresponding putative model complex with cyclosporin A was built based on the complex of *L. donovani* cyclophilin with CsA (3eov) as a template using the program *insightII*. Each model complex was subjected to a very limited energy refinement (100 cycles with the *insightII* Discover Module, steepest descent algorithm).The 3eov CsA binding residues (R78, I80, F83, M84, Q86, G95, T96, A123, N124, A125, G126, Q133, F135, W143, L144, H148) at less than 4 Å from CsA, were used for the superposition. The subsequent analyses of the 3D model complexes and evaluations of the putative interaction with the CsA were performed with the program *insightII*.

### FACS-based approaches

Cell death was assessed by propidium iodide exclusion assay [36]. Briefly, 10^7^ promastigotes or axenic amastigotes from control or CsA treated cultures were washed and resuspended in PBS containing 2 µg/ml of propidium iodide and incubated at room temperature for 15 min in the dark. The stained cells were subjected to FACS analysis (exλ = 488 nm; emλ = 617 nm). 10,000 events were analyzed. For cell cycle analysis, 10^7^ late-log phase promastigotes were washed once with cold PBS and resuspended in pre-chilled 90% methanol in PBS and kept at −20°C overnight. The fixed cells were washed once with cold PBS and then resuspended in propidium iodide staining solution (10 µg/ml PI, 100 µg/ml RNase A in PBS) and incubated at 37°C for 30 min in the dark. The stained cells were subjected to FACS analysis as described above. Cell cycle distribution was calculated by FlowJo (Tree Star, Inc.) using the Dean-Jett-Fox model.

### Morphological analysis

For Giemsa staining, 10^7^ promastigotes or axenic amastigotes were immobilized on poly-L-lysine coated cover slips, fixed with methanol and stained with Giemsa reagent (Sigma) according to the manufacturer's instructions. The stained cells were mounted with Mowiol 4-88 (Sigma) [37] and observed with a Zeiss Axioplan 2 wide field light microscope.

Cells were prepared for scanning electron microscopy as described [38]. Briefly, parasites were washed in PBS, fixed with 2.5% glutaraldehyde in PBS, and treated with 1% OsO_4_. The cells were then dehydrated and critical-point dried (Emitech K850 or Balzers Union CPD30) and coated with gold (Joel JFC-1200 or Gatan Ion Beam Coater 681). Samples were visualized with scanning microscope Joel JM6700 F.

Indirect immunofluorescence staining was performed with 10^7^ promastigotes or axenic amastigotes that were settled on poly-L-lysine coated coverslips and fixed in methanol at -20°C for 5 min. The fixed cells were rehydrated with PBS, and sequentially incubated with a mouse anti-α-tubulin antibody (Sigma, 1∶2500 dilution) and an anti-mouse IgG-rhodamine antibody (Molecular Probes, 1∶250 dilution). Nuclei and kinetoplasts were stained with DAPI and the slides were mounted with Prolong (Molecular Probes).

### Cyclosporin A affinity chromatography

Modified CsA with a primary amine side chain was provided by the Texas A&M Natural Products LINCHPIN Laboratory, Assistant Director Dr. Jing Li [39]. The CsA-amine was coupled to the Affi-Gel®10 resin (Bio-Rad) by reaction with the N-hydroxysuccinimide ester groups of the resin. Briefly, 7.5 µmol of CsA-amine was mixed with 500 µl of Affi-Gel® 10 and incubated at room temperature for 6 hours. The coupling reaction was quenched by removing the CsA-amine and blocking the unreacted Affi-Gel® 10 with 0.2 M ethanolamine. Logarithmic promastigotes were lysed with lysis buffer (50 mM HEPES, 100 mM NaCl, 10% glycerol, 0.5% NP-40 and 1 mM PMSF) followed by sonication on ice (30 s sonication with 15 s pause for 5 min). Insoluble debris was removed by centrifugation. The cleared cell lysate (1 mg protein/ml) was mixed with the CsA-Affi-Gel and incubated at 4°C for 3 hours. Bound proteins were eluted using hot Laemmli buffer. The elution was subjected to 10% SDS-PAGE, stained with SyproRuby®protein gel stain (Invitrogen), and CsA-binding proteins were identified by MS analysis as described [22] and Western blotting.

### Recombinant LmaCyP40 production

*Leishmania major* CyP40 was amplified from *L. major* Friedlin V1 (MHOM/JL/80/Friedlin) genomic DNA using the primers 5′-CTCGAGGGAGGAATGCCGAACACATACTGC-3′ (*XhoI* site and 2 glycine residues are underlined) and 5′-GCGGCCGCAACCCTCACGAGAACATC-3′ (*NotI* site is underlined) and ligated to pGEM-T (Promega). The insert was then released by *Xho*I and *Not*I and ligated into pGEM-HAstrep. The intermediate construct was digested with *BamHI* and *NotI* to release the strep::CyP40 and ligated into pGEX-5X-1 (Amersham Biosciences). The resulting plasmid pGEX-5X-Strep::CYP40 was replicated in *E.coli* BL21. Recombinant GST::strep::CyP40 was induced with 0.2 mM IPTG overnight at room temperature and then purified with GSH-sepharose and strep-tactin sepharose (Fig. S1) using an Äkta Purifier FPLC system (Amersham Biosciences).

### Peptidyl Prolyl *cis/trans* isomerization assay

Measurements were performed according to [40]. Briefly, the peptidyl prolyl *cis/trans* isomerization reaction was initiated by diluting the peptide Abz-Ala-Ala-Pro-Phe-pNA in an anhydrous 0.5 M LiCl/TFE mixture with 35 mM HEPES pH 7.8. Inhibition of PPIase activity was measured by pre-incubating CsA with the enzyme (29.5 nM) for 5 min at 10°C before starting the reaction by the addition of the substrate. Data analysis was performed by single exponential non-linear regression using Sigma Plot Scientific Graphing System.

### Immunoblotting

Parasites (10^8^ cells) were lysed in 1× Laemmli buffer (1×10^9^ cells/ml) and vortexed vigorously for 30 seconds. The lysates were sonicated for 1 min on ice and boiled for 5 min. Soluble fractions were collected as protein extracts after brief centrifugation. Twenty microliters of samples (equivalent to 2×10^7^ cells) were separated by 10% SDS-PAGE and then transferred to PVDF membrane. Mouse anti-LPG antibody (clone CA74E, 1∶5000 dilution) [41], mouse anti-A2 antibody (clone C9, 1∶200 dilution) [42], rabbit polyclonal anti-CyP40 (established using recombinant strep::CyP40 as antigen, 1∶5000 dilution, Eurogentec), and appropriate HRP-conjugated secondary antibodies were used to probe the membrane using dilutions of 1∶10000 and 1∶50000, respectively, and signals were revealed by SuperSignal ECL from ThermoFisher.

## Results

### The *Leishmania* genome encodes for a large cyclophilin protein family

**Figure 1:**
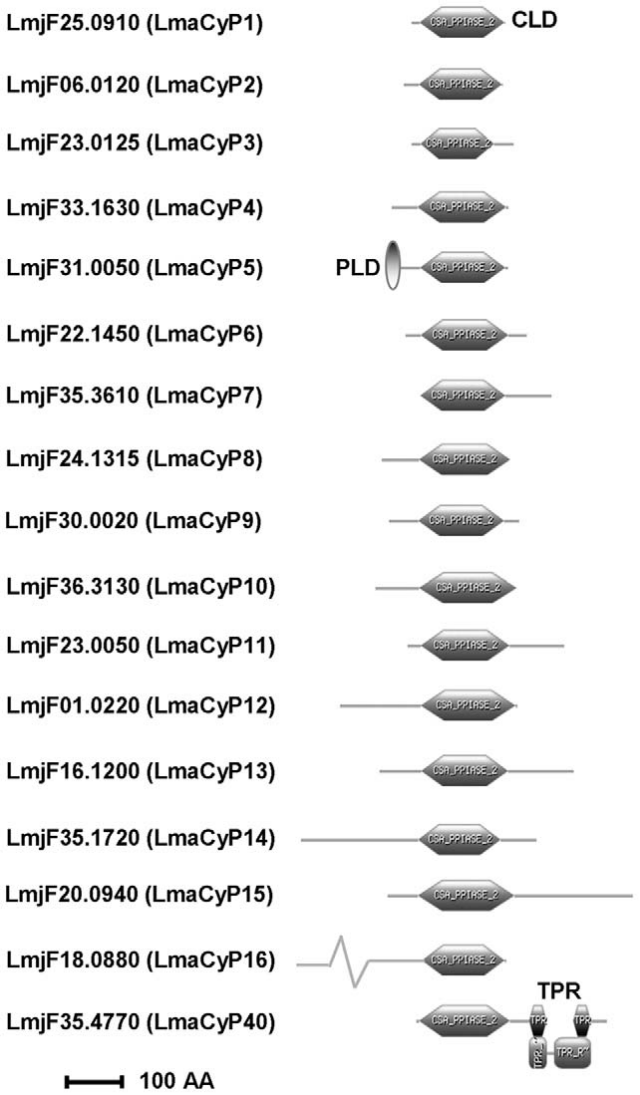
Diagram representing the *L. major* CyP-like proteins. 17 CyP-like proteins are annotated in the *L. major* genome database (LmaCyPs). The cyclophilin-like domain (CLD) and other domains were identified with ScanProsite. Most LmaCyPs are characterized by parasite-specific N- and C-terminal extensions. Additional functional domains are identified in two LmaCyPs, LmjF31.0050 (LmaCyP5) and LmjF35.4770 (LmaCyP40), containing a prokaryotic lipid attachment domain (PLD) and tetratricopeptide repeat domains (TPR), respectively. The bar represents 100 amino acids.

**Figure 2:**
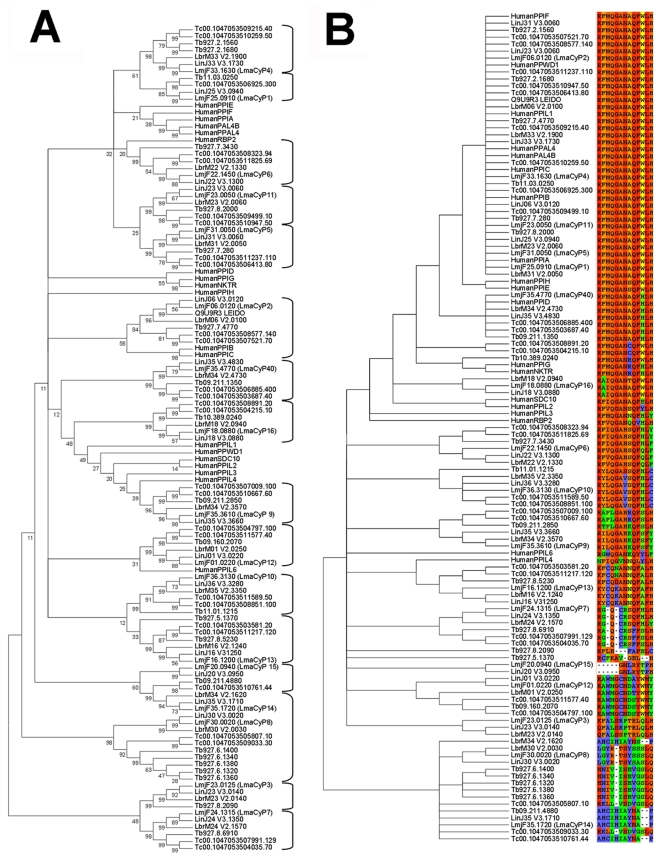
Bioinformatics analysis of the *L. major* CyP-like protein family. (*A*) *Neighbor-Joining tree (500 bootstrap replicates) of the 118 CyP-like proteins*. CyP proteins were identified by PSI-Blasting Human PPIaseA against Human, *L. major*, *L. infantum*, *L. braziliensis*, *T. brucei*, and *T. cruzi* genomes. Multiple sequence alignment was performed with T-Coffee and fed into the MEGA4 software package. Numbers on nodes indicate bootstrap support. (*B*) *UPGMA clustering of CyP functional residues*. Positions corresponding to the CsA binding sites (as defined on the Human PPIaseA) are displayed on the figure along with the CyP-like protein they originate from. The UPGMA clustering shows groups of putatively identical binding sites.

PPIases are classified according to the binding of the inhibitors cyclosporin A (CsA) and FK506 in two major protein families, cyclophilins (CyPs) and FK506 binding proteins (FKBPs), respectively [43]-[45]. A third PPIase family is represented by PpiC/parvulin-like proteins implicated in proline-directed phosphorylation [46], [47]. Based on the presence of a conserved CyP-type PPIase signature sequence, termed cyclophilin-like domain, CLD, (Prosite accession number: PS50072, FY-xx-STCNLVA-x-FV-H-RH-LIVMNS-LIVM-xx-F-LIVM-x-Q-AGFT), the *Leishmania major* genome encodes for 17 cyclophilin-like proteins (LmaCyPs), five FKBP-like LmaFKBPs, and two PpiC/parvulin-like LmaPPICs (Fig. 1 and Table 1), all of which are conserved in the *L. infantum* and *L. braziliensis* genomes (Fig. 2). According to the current nomenclature [2], the LmaCyPs were distinguished by numbering from the smallest to the highest predicted molecular weight (Table 1).

**Table 1:** PPIase domain containing proteins in *Leishmania major*.

Type of PPIase	GeneDB ID	Proposed annotation	Size		Positions (aa) of the PPIASE domains and other features	Remarks
			aa	kDa		
**CyP-like**	**LmjF25.0910**	*LmaCyP1*	**177**	**18.8**	**CSA_PPIASE (17–176)**	**1**
	**LmjF06.0120**	*LmaCyP2*	**187**	**20.3**	**CSA_PPIASE (30–186)**	**1**
	**LmjF23.0125**	*LmaCyP3*	**192**	**20.3**	**CSA_PPIASE (18–156)**	**1**
	**LmjF33.1630**	*LmaCyP4*	**220**	**24.0**	**CSA_PPIASE (50–216)**	**2 N**
	**LmjF31.0050**	*LmaCyP5*	**229**	**24.6**	**CSA_PPIASE (64–223), PROKAR_LIPOPR (1–23)**	**2 N, 3**
	**LmjF22.1450**	*LmaCyP6*	**229**	**25.2**	**CSA_PPIASE (30–194)**	**1**
	**LmjF35.3610**	*LmaCyP7*	**247**	**25.5**	**CSA_PPIASE (1–160)**	**2 C**
	**LmjF24.1315**	*LmaCyP8*	**242**	**26.4**	**CSA_PPIASE (71–242)**	**2 N** *
	**LmjF30.0020**	*LmaCyP9*	**245**	**27.5**	**CSA_PPIASE (56–216)**	**2 N, C**
	**LmjF36.3130**	*LmaCyP10*	**266**	**28.8**	**CSA_PPIASE (83–265)**	**2 N** *
	**LmjF23.0050**	*LmaCyP11*	**295**	**31.4**	**CSA_PPIASE (27–193)**	**2 C**
	**LmjF01.0220**	*LmaCyP12*	**335**	**36.1**	**CSA_PPIASE (150–330)**	**2 N**
	**LmjF16.1200**	*LmaCyP13*	**366**	**38.6**	**CSA_PPIASE (80–243)**	**2 N, C** *
	**LmjF35.1720**	*LmaCyP14*	**444**	**48.5**	**CSA_PPIASE (222–376)**	**2 N** * **, C**
	**LmjF20.0940**	*LmaCyP15*	**462**	**49.0**	**CSA_PPIASE (58–238)**	**2 N, C**
	**LmjF18.0880**	*LmaCyP16*	**1020**	**108.1**	**CSA_PPIASE (863–1017)**	**2 N**
	**LmjF35.4770**	*LmaCyP40*	**354**	**38.4**	**CSA_PPIASE (7–174), TPR (210–243; 257–290; 291–324)**	**3**
**FKBP-like**	**LmjF22.1430**	*LmaFKBPL1*	**111**	**11.8**	**FKBP_PPIASE (23–111)**	**1**
	**LmjF36.0230**	*LmaFKBPL2*	**109**	**11.9**	**FKBP_PPIASE (19–109)**	**1**
	**LmjF10.0890**	*LmaFKBPL3*	**159**	**17.2**	**FKBP_PPIASE (49–135)**	**2 N** * **, C** *
	**LmjF19.0970**	*LmaFKBPL4*	**202**	**22.8**	**FKBP_PPIASE (81–168)**	**2 N**
	**LmjF19.1530**	*LmaFKBPL5*	**432**	**47.6**	**FKBP_PPIASE (56–144), TPR (335–402)**	**2 N, 3**
**Parvulin-like**	**LmjF07.1030**	*LmaPPICL1*	**115**	**12.5**	**PPIC_PPIASE (3–115)**	**1**
	**LmjF22.0530**	*LmaPPICL2*	**440**	**46.5**	**PPIC_PPIASE (95–146), FHA (313–440)**	**2 N, 3**

**Footnote.** PPIase like proteins were retrieved by Blast analysis of the PPIase signature sequences in GeneDB (www.genedb.org). Prosite accession numbers of the domains are: CSA_PPIASE: PS50072; FKBP_PPIASE: PS50059; PPIC_PPIASE: PS01096; PROKAR_LIPOPR: PS51257; TPR: PS50005; FHA: PS50006. Sequences flanking the PPIase domain were analyzed by Blast search in NCBI (blast.ncbi.nlm.nih.gov/Blast.cgi). 1, a single PPIase domain without any significant N- or C-terminal sequence extensions; 2, presence of parasite-specific N-terminal (N), C-terminal (C), or both extensions; 3, presence of additional functional domains;

*, represents the extension is unique to *Leishmania*;

#, Protein function with experimental support published [60], [61].

Based on length and domain structure, three types of *L. major* cyclophilins (LmaCyPs) can be distinguished. A first group of four proteins (LmaCyP1–3, 6) is characterized by a single CsA-binding domain without any significant N- or C-terminal sequence extensions (Fig. 1 and Table 1). A second group of 11 proteins shows significant (50 or more amino acids) N-terminal (LmaCyP4, 5, 8, 10, 12, 16), C-terminal (LmaCyP7, 11), or both N- and C-terminal extensions (LmaCyP9, 13–15). These extensions are unique and not conserved in human CyPs, but are mostly conserved across other trypanosomatids, including *L. infantum*, *T. brucei* and *T. cruzi*. Exceptions are the C-terminus of LmaCyP13 and the N-termini of LmaCyP8, 10, and 14, which are unique to *Leishmania* suggesting highly parasite specific functions absent in *Trypanosoma*. Finally, two LmaCYPs are characterized by the presence of additional functional domains, including LmaCyP5 containing a conserved prokaryotic lipid attachment domain (PLD, prosite access number PS5125), and LmaCyP40, the cyclophilin-40 homolog containing two tetratricopeptide repeat domains (TPR, prosite accession number PS50005) known to interact with HSP90 [48]–[51].

### Bio-informatics characterization of the LmaCyP protein family

We investigated the relationship between human and trypanosomatid CyPs by multiple alignment and cluster analysis using the sequence of the conserved CLD or the functional residues implicated in PPIase function and CsA binding. The clustering tree obtained for the CLD demonstrates that all LmaCyPs have conserved homologs in *L. infantum, L. braziliensis, T. brucei*, and *T. cruzi*, which cluster together with highly significant bootstrap values (Fig. 2A). All LmaCyPs have one homologue in the other *Leishmania* or *Trypanosoma* species, with the exception of LmaCyP5, which underwent expansion in the *T. brucei* genome with five sequentially arranged copies of the gene. It is interesting to speculate that the expansion of the only cyclophilin family member that contains a conserved lipid binding domain may be a reflection of the *T. brucei* biology, with a potential role for example for the expression of abundant gpi-anchored VSG proteins [52].

Many of the nodes support the existence of CyP subclasses across the trypanosomatids with a significant bootstrap value. In contrast, the nodes that cluster these subclasses together with their human homologues have only poor bootstrap support. This observation suggests that the various classes of CLDs encountered in trypanosomatid cyclophilins are quite distinct from one subclass to another and to their human counterparts. Substantial conservation however was observed in the cluster analysis performed with the functional CyP residues implicated in PPIase function and CsA binding (Fig. 2B). For instance, eight human CyPs and five LmaCyPs are clustering together showing a complete conservation of the canonical signature sequence characteristic for the human CsA-binding protein PPIA (Fig. 2B and Table 2). This represents a significant conservation when considering that the overall CLD domain is only 64% conserved between the *Leishmania* and Human CyPs. These results indicate that a subset of *Leishmania* CyPs are likely functionally conserved and implicated in PPIase function, while other, less conserved LmaCyPs may carry different enzymatic activities.

**Table 2:** Conservation of LmaCyP functional sites compared to human CyPA.

	HsCyPA CsA functional residues												
Residue	R	F	M	Q	G	A	N	A	Q	F	W	L	H
Position	55	60	61	63	72	101	102	103	111	113	121	122	126
*LmaCyP1*													
*LmaCyP2*													
*LmaCyP3*	L53	E58	A59		D71	R93	P94	T95	R103	L105	Q113		
*LmaCyP4*													
*LmaCyP5*													
*LmaCyP6*			V89				H132	S133			Q151		F156
*LmaCyP7*		I49	L50				G97	I98	D106	G108	T119		
*LmaCyP8*		G132			P144	C167	R168	S169			H191		
*LmaCyP9*	T108	I113	D114		R121	T153	S154	Y155	S163	S165	S173		Q178
*LmaCyP10*		Y158	L159				V202	S203			H222		C227
*LmaCyP11*													
*LmaCyP12*		A225	W226	M228		C267		D269	A227	Y279		M228	Y292
*LmaCyP13*		Y133	C134		K145			N180			A200	F201	
*LmaCyP14*	A272	H277	C278	I280	D290	I316	A317	Y318	N326	A328	S333	G334	P338
*LmaCyP15*		S117	V118	E120	F130	G167		L169	R178	Y180	R192	H193	F198
*LmaCyP16*		A902	I903										
*LmaCyP40*											H131		

**Footnote**. The functional amino acid residues implicated in PPIase catalytic activity and CsA binding of human CyPA (HsCyPA) (NCBI accession no. NP_066953) are shown [73], [74]. LmaCyPs were aligned against the human CyPA domain by ClustalX 2.0 with matrix Blosum62. Amino acid changes of LmaCyPs compared to human CyPA are shown.

In conclusion, our analysis reveals a large *Leishmania* CyP protein family suggesting an important role of PPIases in parasite biology, and identifies unique sequence elements in the LmaCyP CsA-binding domains that may be exploitable for drug development. Identification of 5 out of 17 LmaCyPs with a highly conserved CsA binding motif strongly suggests inhibitor-binding to multiple LmaCyPs with potentially important consequences on the biological functions of these proteins and *Leishmania* infectivity. In the following we investigate this possibility studying the effects of CsA on *L. donovani* promastigotes and amastigotes in culture.

### CsA treatment interferes with parasite growth *in vitro*

**Figure 3:**
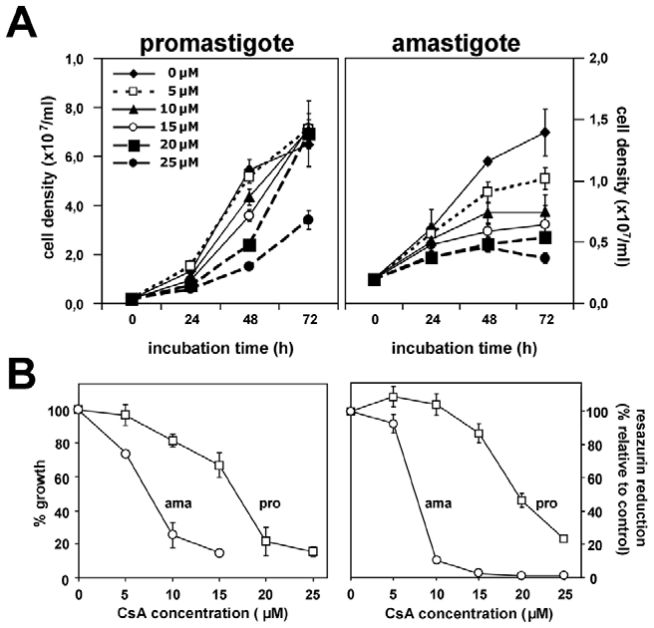
CsA inhibits *L. donovani in vitro* growth. (*A*) Parasites were treated for up to 48 hours with CsA at the concentrations indicated at 26°C and pH 7.4 for promastigotes, or 37°C and pH 5.5 for amastigotes. Cell density of the samples was estimated using CASY cell counter and expressed in cell density per milliliter. (*B*) *Left panel, growth assessment*. Logarithmic promastigotes (pro, □) and axenic amastigotes (ama, ○) were incubated for 48 hours with CsA at the indicated concentrations. Cell density of the samples was estimated using hemocytometer and expressed in % of growth compared to solvent treated controls. *Right panel, cell proliferation and viability assay*. Promastigotes and axenic amastigotes were treated as detailed in (A). 20 µl of CellTiter-Blue solution was added to the cells after 48 hours of CsA treatment, and the assay was further incubated for 4 hours at 37°C. Resazurin reduction was expressed in % of fluorescence compared to solvent treated cells control. Results are representative of three quadruplicate experiments with mean ± S.D. indicated by the error bars.

CsA has been previously shown to reduce the intracellular growth of *L. major* amastigotes [13], [14]. To further elucidate the mechanisms underlying this inhibition, we investigated the effects of CsA treatment on cultured *L. donovani* promastigotes and axenic amastigotes. Log-phase parasites from both stages (2×10^6^/ml) were cultured in medium containing either ethanol (vehicle) or CsA at concentrations ranging from 5 to 25 µM, and incubated at 26°C, pH 7.4 (promastigote) or 37°C, pH 5.5 (amastigote) for 48 hours. At the time points indicated, the cells were fixed and counted microscopically or processed for CellTiter-Blue assay to test for proliferation. CsA-treated promastigotes showed a dose-dependent, progressive reduction of growth with an IC50 at 48 hours between 15 and 20 µM and a more than 5-fold decrease in growth at the highest inhibitor concentration compared to the control (Fig. 3A and B, left panels). Growth reduction was associated with a strong inhibition of resazurin reduction indicating reduced cell proliferation or cell viability (Fig. 3B, right panel). CsA-mediated growth reduction was reversible, as parasite growth resumed normally after removal of the drug after 48 hours of treatment (data not shown). Likewise, CsA had a striking effect on the growth of *L. donovani* axenic amastigotes. The parasites showed substantially higher susceptibility to CsA at this stage with an IC50 between 5 and 10 µM (Fig. 3A, right panel, and Fig. 3B, left panel), and strongly reduced resazurin reduction (Fig. 3B, right panel). Together, our data demonstrate that CsA interferes with the *in vitro* growth of both *L. donovani* promastigotes and axenic amastigotes. In the following we used FACS-based approaches to investigate the mechanisms underlying this growth defect.

### CsA shows a stage-specific effect on *L. donovani* viability

**Figure 4:**
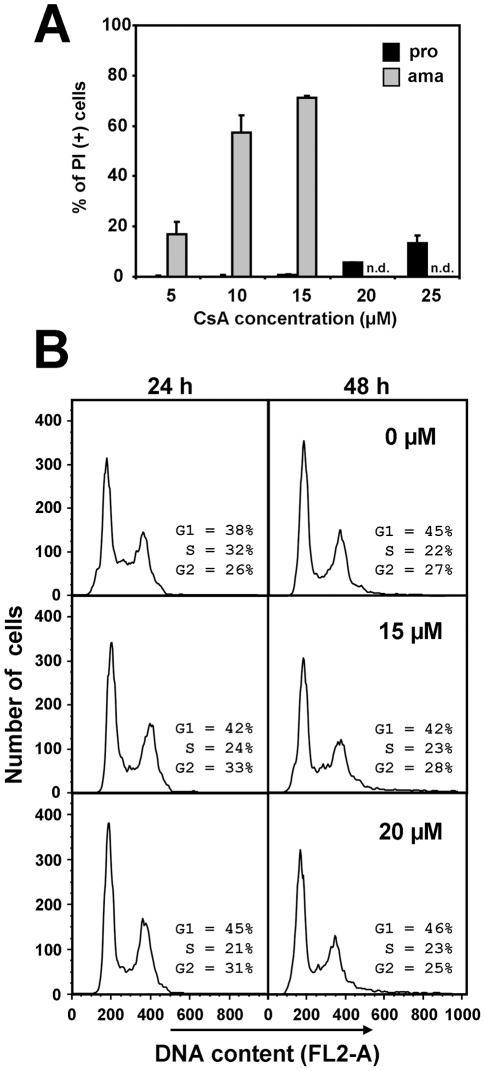
CsA affects *L. donovani* viability and proliferation. *(A) FACS analysis*. Logarithmic promastigotes (black bars) and axenic amastigotes (grey bars) were incubated for 48 hours with the indicated CsA concentrations. The cells were then washed once with PBS, stained with 2 µg/ml of PI and analyzed by FACS. Proportion of dead parasites is expressed in % of PI positive (+) stained cells after subtracting the background of solvent treated cells control. The error bars represent the mean±S.D. of four independent experiments. *(B) Cell cycle analysis*. CsA-treated promastigotes were fixed in cold 90% methanol and stained with propidium iodide for cell cycle analysis. The stained cells were subjected to FACS analysis (exλ = 488 nm/emλ = 617 nm). 10,000 events were analyzed and cell cycle distribution was calculated with the model Dean-Jett-Fox using the FlowJo Software package (Tree Star, Inc.). At least two independent experiments were performed and representative results are shown.

To elucidate the mechanisms of CsA-mediated growth inhibition, we first investigated the effects of CsA on the viability of treated promastigotes and axenic amastigotes using a propidium iodide (PI) exclusion assay [36]. The percentages of PI positive, dead promastigotes and axenic amastigotes after 48 hours of CsA treatment was revealed by FACS analysis. Promastigotes did not show any significant increase in PI positive cells after incubation with CsA ranging from 5 to 15 µM (Fig. 4A), and more than 80% of cells were viable even at 25 µM CsA. In contrast, the proportion of PI positive axenic amastigotes increased dramatically with increasing CsA concentration, with a 4-fold decrease in cell viability at only 10 µM CsA (Fig. 4A). Thus, the decrease in cell number of CsA-treated promastigotes results from a slow-down in proliferation rather than parasite killing.

This result was further confirmed by cell cycle analysis. Late-log phase promastigotes were fixed with 90% methanol in PBS, stained with PI, and cell cycle phase distribution was determined by FACS analysis. Treatment of the parasites with 15 µM and 20 µM CsA did not affect the cell cycle distribution (Fig. 4B), suggesting that inhibition of parasite proliferation results from a non-synchronous slow-down in cell cycle progression.

### CsA treatment induces morphological changes in *L. donovani* promastigotes without induction of amastigote gene expression

**Figure 5:**
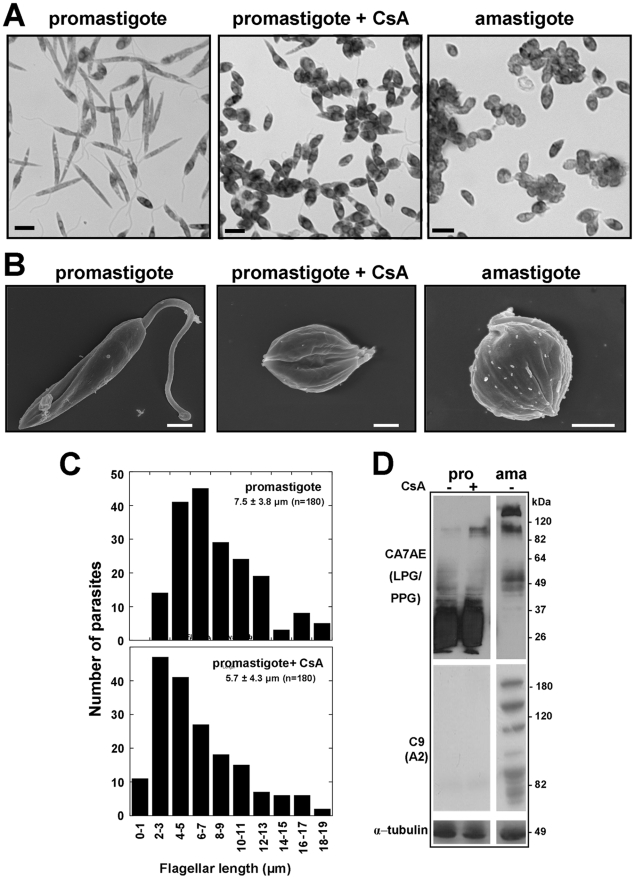
CsA-treated *L. donovani* promastigotes show altered morphology. Promastigotes were incubated with 0.15% ethanol or 15 µM (B, C) or 20 µM (A) CsA at 26°C, pH 7.4 for 72 hours. Axenic amastigotes were prepared as described in experimental procedure. 10^7^ cells were fixed with either methanol for Giemsa staining (*A*), or 2.5% glutaraldehyde for scanning electron microscopy (*B*). The bar corresponds to 1 µm (B) and 5 µm (A). Two independent experiments were performed and representative fields are shown. (*C*) *Flagellum length measurement*. CsA-treated and solvent treated cells *L. donovani* promastigotes were fixed in methanol and stained with anti-tubulin monoclonal antibody. Flagellum length was measured from a total of 180 cells each for control and CsA-treated samples. Only cells with a single flagellum that was completely visible and fully in focus were taken into account. Samples were observed with a DMR Leica microscope and images were captured with a Cool Snap HQ camera (Roper Scientific). Images were analysed using the IPLab Spectrum 3.9 software (Scanalytics & BD Biosciences) and flagellum length was measured using ImageJ (NIH). (*D*) *Immunoblot analysis of CsA treated parasites*. Parasites were treated with solvent or 15 µM CsA for 72 hours, lysed in 1× Laemmli buffer, and lysates equal to 2×10^7^ cells were analyzed by immunoblotting. Promastigote specific marker LPG (upper), amastigote specific marker A2 (middle) and α-tubulin (lower) were analyzed. Two independent experiments which gave identical results were performed.

CsA-treatment of promastigote cultures induced a striking effect on parasite morphology. We documented these alterations by microscopic analysis using Giemsa staining and scanning electron microscopy. Treatment of promastigotes with 10 to 20 µM CsA induced morphological changes reminiscent of axenic amastigotes, including increased aggregate formation (Fig. 5A), oval cell shape (Fig. 5B), and shortened and retracted flagella (Fig. 5C).

The CsA effects on *L. donovani* promastigotes are reminiscent to parasites treated with the HSP90 inhibitor geldanamycin, which results in amastigote differentiation [53]. We evaluated the effect of CsA on the differentiation state by following the expression of two markers, the promastigote specific surface glycoconjugates lipophosphoglycan (LPG) [54], which is lost during amastigote differentiation, and the A2 protein, which is induced during the pro- to amastigote conversion [42], [55]. Logarithmic promastigotes were incubated with vehicle alone (0.15% ethanol) or 15 µM CsA at 26°C, pH 7.4 for 72 hours, and the expression profile was compared to axenic amastigotes by Western blotting using monoclonal anti-lipophosphoglycan antibody CA7AE [41] and anti-A2 antibody C9 [42]. Despite the amastigote-like morphology, CsA-treated promastigotes maintain expression of LPG, comparable to the level of solvent treated cells promastigotes, and do not show induction of the amastigote marker protein A2 (Fig. 5D). CsA treatment of promastigotes at pH 5.5 did not result in further differentiation as judged by morphology and expression of LPG, nor did it have an effect on parasite viability (data not shown). These results demonstrate that unlike geldanamycin, CsA induces morphological features similar to amastigotes without inducing the appropriate expression profile.

### Investigation of the stage-specific mechanisms underlying CsA inhibition

**Figure 6:**
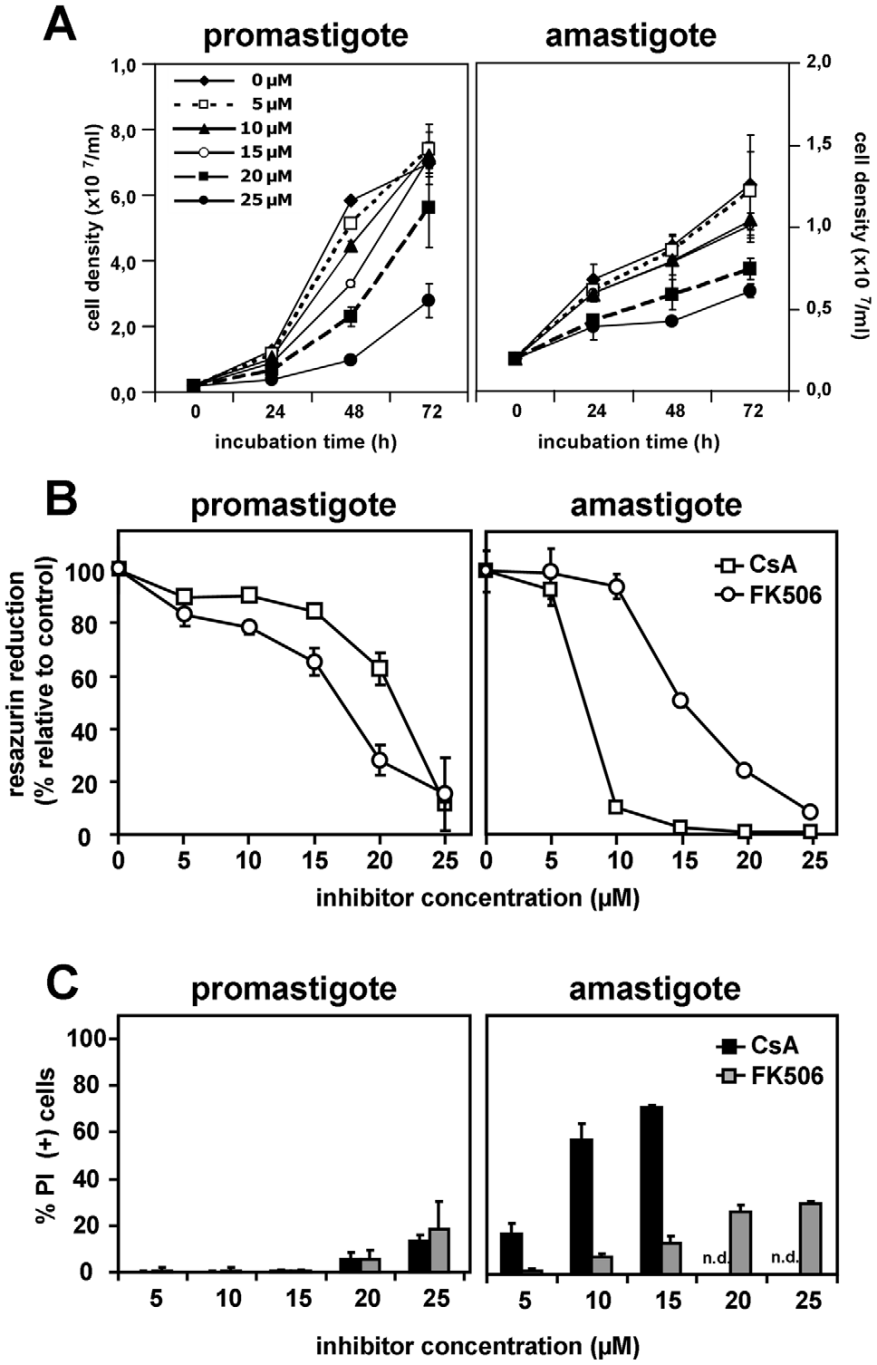
The stage-specific effects of CsA occur through distinct mechanisms. Parasites were treated for 48 hours with either CsA or FK506 at the concentrations indicated at 26°C and pH 7.4 for promastigotes, or 37°C and pH 5.5 for amastigotes. (*A*) Cell density of the samples was estimated using CASY cell counter and expressed in cell number per milliliter, after 24 to 48 hours treatment. (*B*) Cell proliferation was measured using CellTiter-Blue cell viability assay by following resazurin reduction, which is expressed in % of fluorescence compared to solvent treated cells control. (*C*) Cell death was measured by propidium iodide staining and FACS analysis as detailed in legend of Fig. 3. Results of (*A*) are representative of three quadruplicate experiments with mean ± S.D represented by the error bars. Three independent experiments were performed for (B) with the error bars representing ±S.D.

CsA exerts its inhibitory action through binding of CyPs and inactivation of the cellular phosphatase calcineurin by CsA/CyP complexes [8], [56]. In the following, we used the unrelated calcineurin inhibitor FK506 to analyze if the CsA effects on the parasite are mediated through inhibition of this phosphatase, a test that has been previously applied on *Leishmania* [57]. Log-phase promastigotes and axenic amastigotes (2×10^6^/ml) were cultured for 48 hours in medium containing either ethanol (vehicle) or FK506 at concentrations ranging from 5 to 25 µM, and incubated at 26°C, pH 7.4 (promastigote) or 37°C, pH 5.5 (amastigote). FK506 treatment of promastigotes induced morphological changes similar to CsA treated parasites, and strongly reduced *in vitro* growth and cell proliferation in a dose-dependent manner (Fig. 6A and B, left panels, and data not shown). Like CsA, FK506 did not significantly affect promastigote cell viability at the lower drug concentrations (Fig. 6B, left panel). In contrast, FK506 treatment of axenic amastigotes did not reproduce the CsA effects. First, as judged by proliferation and viability assay, amastigotes were more resistant to FK506, with an IC50 between 15 and 20 µM, compared to ca. 7 µM for CsA (Fig. 6B, right panel). Second, unlike CsA, FK506 did not induce massive cell death in amastigotes even at the highest concentration (Fig. 6C, right panel). These data show that CsA and FK506 have different effects on *L. donovani* axenic amastigotes, which may be due to either stage-specific differences in inhibitor uptake or distinct intracellular cellular targets.

### CsA treatment reduces *L. donovani* thermotolerance

**Figure 7:**
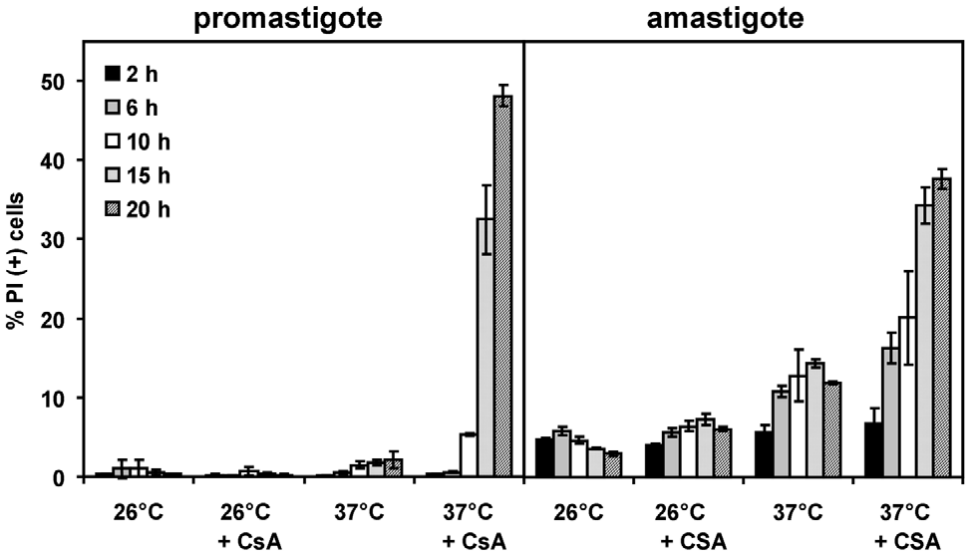
CsA affects *L. donovani* thermotolerance. Axenic amastigotes and promastigotes were either treated with solvent or 15 µM CsA, and incubated at either 26°C or 37°C. At the time points indicated, aliquots of the cells were stained with propidium iodide and analyzed by FACS. The proportion of dead parasites is expressed in % of PI positive (+) cells. Three independent experiments were performed, and one representative triplicate experiment is shown. The error bars represent ±S.D.

Based on previously published observations, *Leishmania* CyPs may have important amastigote-specific chaperone functions and participate in protein disaggregation [20]. We tested if CsA treatment affects thermotolerance of promastigotes and amastigotes following the number of propidium iodide stained, dead parasites as a read out. Log-phase promastigotes or amastigotes were treated with 15 µM CsA and parasites were simultaneously incubated for various time periods at either 26°C or 37°C. As expected, CsA treated amastigotes showed increased cell death in the presence of CsA during the 20 hours time course experiment (Fig. 7, right panel). Significantly, CsA-treatment of amastigotes at 26°C completely abrogated the toxic effect of the inhibitor. This data shows that CsA-mediated amastigote killing is temperature dependent. We confirmed this result using the complementary set up, incubating CsA-treated promastigotes at high temperature. Just like amastigotes, CsA-treated promastigotes underwent cell death as soon as 10 hours after temperature shift (Fig. 7, left panel). CsA alone or heat shock alone had no significant effect on promastigote viability. Thus, CsA affects thermotolerance of both the promastigote and amastigote stages.

### Identification of CsA-binding *Leishmania donovani* cyclophilins

**Figure 8:**
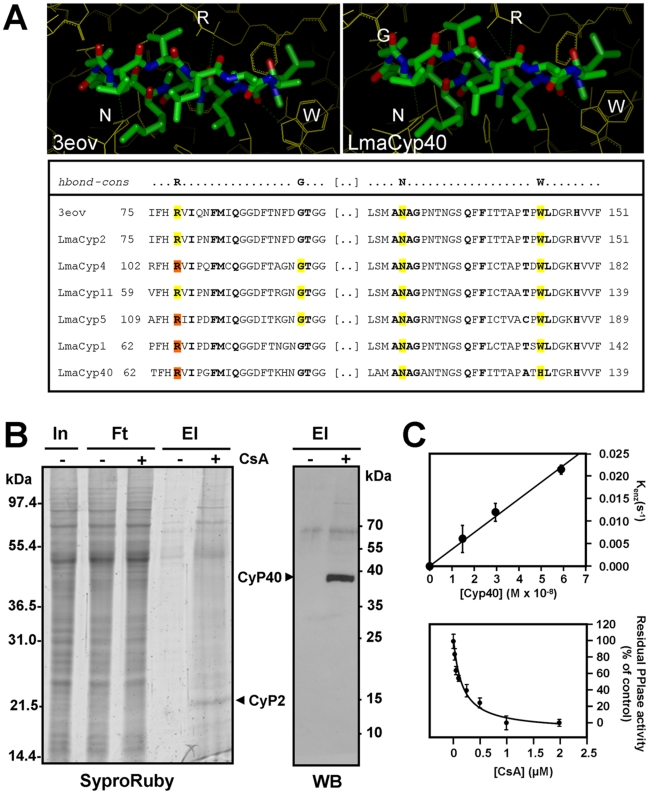
*Leishmania* cyclophilin 40 is a target for CsA. (*A*) *Structural modelling*. Cyclosporin (CsA) binding pockets of *L. donovani* cyclophilin CyP2 (PDB code: 3eov, left upper panel) and *L. major* CyP40 in the presence of the CsA ligand (model complex, right upper panel) are shown. Cyclophilin residues are coloured in yellow and CsA is coloured by atom type. H-bonds are displayed as green dotted lines. The lower panel shows a multiple sequence alignment of the CsA binding regions of *L. donovani* CyP2 (PDB code: 3eov) and the six *L. major* cyclophilins analyzed. The residues in close proximity to the ligand are shown in bold. Yellow and orange filling identifies residues forming respectively one or two hydrogen bonds with CsA atoms. (*B*) *Affinity chromatography and Western blotting*. Total protein extracts obtained from logarithmic *L. donovani* promastigote cultures were incubated with resin alone (−), or resin coupled with CsA (+), bound proteins were analyzed by SDS-PAGE and SyproRuby staining, and identified by MS analysis (left panel, CyP2), or Western blotting using polyclonal anti-LmaCyP40 antibody (right panel). In, input; Ft, flow through; El, eluate. (*C*) *PPIase assay*. The *cis/trans* isomerization activity of recombinant *Leishmania major* GST::Strep::CyP40 was performed using Abz-Ala-Ala-Pro-Phe-pNA as substrate. Determination of k_cat_/K_m_ (upper panel) was performed by evaluation of the linear dependency of k_enz_ from the concentration of GST::Strep::CyP40. Each data point represents the mean of three independent measurements. The lower panel shows inhibition of *Leishmania major* GST::Strep::CyP40 peptidyl prolyl *cis/trans* isomerase activity by CsA. PPIase activity was measured at increasing amounts of CsA using the substrate Abz-Ala-Ala-Pro-Phe-pNA.

The effect of CsA on parasite thermotolerance primed us to investigate the potential interaction between this inhibitor and LmaCyP40, a bifunctional cyclophilin that has both PPIase and co-chaperone function and interacts with members of the HSP protein family through TPR domains [58]. We first used a structural approach applied on six leishmanial cyclophilins selected for their similarity to the cyclosporin A binding pocket of human orthologs. We built the corresponding model complexes with CsA and evaluated their geometric fit and ability to establish inter-molecular hydrogen bonds with the ligand. The experimentally identified CsA binding residues of the *L. donovani* cyclophilin (3eov) and the putative binding residues of the *L. major* 3D model complexes, including the one for LmaCyP40, are highly conserved (Fig. 8A). All models, even if built on different templates, display a root mean square deviation of less than 0.6 angstrom on the CsA binding residues of the experimentally determined complex structure. Consequently, all models can accommodate the CsA ligand with no molecular clash and the hydrogen-bonding pattern is conserved with respect to the experimental structure (Fig. 8A, lower panel). Furthermore, manual inspection of the model complexes revealed a good geometric complementarity between the protein and the ligand. All these evidences support the hypothesis that these *L. major* cyclophilins, including LmaCyP40, are good candidates for CsA binding.

We confirmed binding of the CsA ligand to LmaCyP40 by studying the proposed interaction by affinity chromatography using CsA-loaded resin. *L. donovani* promastigote extracts were incubated with the resin and bound proteins were separated by SDS-PAGE. One major band, specifically retained on the CsA-resin, was revealed by fluorescent protein gel staining, and identified as CyP2 by MS analysis (Fig. 8B, left panel, and Dataset S1). Western blot analysis of the gel revealed cyclophilin 40 (Fig. 8B, right panel), thus confirming the CsA-CyP40 interaction suggested by the structural modelling.

We next analyzed the biochemical characteristics of the LmaCyP40-CsA interaction using GST::Strep::CyP40 purified from recombinant bacteria (Fig. S1). We first determined the k_cat_/K_m_ of *Leishmania major* GST::Strep::CyP40 PPIase activity by evaluating the linear dependency between k_enz_ and enzyme concentration ranging from 14.7 to 59 nM. The catalytic efficiency of *Leishmania major* GST::Strep::CyP40 for Abz-Ala-Ala-Pro-Phe-pNa was found to be k_cat_/K_M_ =  (3.725±0.16)×10^5^ M^−1^ s^−1^ (Fig. 8C, upper panel). We then tested direct inhibition of the LmaCyP enzymatic activity by CsA using the substrate Abz-Ala-Ala-Pro-Phe-pNA and increasing amounts of inhibitor. The IC50 value of CsA was determined to be 162±46 nM CsA (Fig. 8C, lower panel) and thus similar to human CyP40 with an IC50 value of 195 nM [59].

## Discussion

The leishmanicidal activity of CsA has been first demonstrated in *L. tropica* infected BALB/c mice, which showed a dose-dependent inhibition of parasite burden and reduction in lesion formation [12]. This anti-parasitic activity has been subsequently confirmed for *L. major* in mouse and macrophage infection assays, and various modes of CsA action have been proposed [13], [14], [57]. The observation that CsA has no overt anti-microbial activity against *L. major* promastigotes in culture, but efficiently kills amastigotes in infected macrophages, provided support to the idea that the toxic effect of CsA on intracellular parasites depends on inhibition of host rather than *Leishmania* CyPs. This hypothesis was further supported by findings showing that the phosphatase calcineurin, the prime target of the inhibitory CsA/CyP complex, is expressed at very low levels and is not recognized by *Leishmania* LmaCyP19 (corresponding to LmaCyP1 according to our nomenclature), although this protein efficiently bound CsA [60], [61]. In contrast to these previous reports, our data provide several lines of evidence for a direct action of CsA on *Leishmania* CyPs.

A first line of evidence resulted from the bio-informatics analysis and structural modeling of *Leishmania* CyPs. Blast search of the *L. major* and *L. infantum* genome databases (www.genedb.org) identified a surprisingly large family of 17 CyP-like proteins in these protozoan, compared to yeast, Drosophila, and human with 8, 14 and 19 CyPs, respectively (Table 1, Fig. 2) [62]–[64]. Multiple sequence alignment of trypanosomatid and human CyPs, cluster analysis of the functional residues implicated in PPIase catalytic activity and CsA binding of the CLD, and structural modelling revealed the presence of six *Leishmania* CyPs that showed conservation of the functional residues (Table 2, Figs. 2 and 8A) and were predicted to form a complex with CsA. This remarkable conservation indicates that multiple *Leishmania* CyPs are likely binding to CsA, a fact that we subsequently confirmed by affinity chromatography and Western blotting, revealing direct interaction of the inhibitor with *Leishmana* CyP2 and CyP40 (Fig. 8 B).

The effects of CsA on *L. donovani* promastigotes and axenic amastigotes further support this possibility and provided a second line of evidence for a direct action of CsA on *Leishmania* CyPs *in vitro*. We showed that inhibitor treatment of *L. donovani* promastigotes leads to dose-dependent, reversible inhibition of proliferation (Figs. 3A and B), without significant effects on cell viability (Fig. 4A) and cell cycle distribution (Fig. 4B). These results confirmed previous observations that CsA does not exert a toxic effect on *Leishmania* promastigotes, but revealed a strong effect on promastigote *in vitro* growth that escaped previous analysis, likely due to the lower CsA concentration (4 µM) used in these studies [13], [14]. In contrast to promastigotes, CsA showed a direct toxic effect on *L. donovani* axenic amastigotes with more than 50% of parasite death in the presence of 10 µM inhibitor (Fig. 4A). This result demonstrates for the first time that the observed anti-leishmanial effect on intracellular amastigotes in mouse and macrophage infection [13], [14], [57] may rely mainly on direct inhibition of parasite CyPs by CsA, although a participation of host CyPs can not be excluded. We further investigated the mechanisms underlying the stage-specific effects of CsA using the unrelated antifungal macrolide inhibitor FK506. FK506 binds to FKBPs, a second class of PPIases (Table 1), which similar to the CsA/CyP complexes inhibit calcineurin [8]. FK506 treatment reproduced the effects observed in CsA-treated promastigotes, suggesting inhibition of calcineurin as one of the mechanisms underlying the observed growth defect of this parasite stage (Fig. 6). To our surprise, unlike CsA, FK506 did not exert a toxic effect on axenic amastigotes at concentrations between 5 and 15 µM (Fig. 6B), a fact previously observed in intracellular *L. major* amastigotes [57]. These data indicate that the toxic effect of CsA on amastigotes occurs likely through calcineurin-independent mechanisms, which may be directly linked to inhibition of stage-specific enzymatic functions of *Leishmania* CyPs.

Cyclophilins are protein chaperones with PPIase activity, which catalyzes the *cis-trans* isomerization of peptidyl-prolyl bonds, affecting stability, activity, and localization of client proteins [2], [65]. Thus, inhibition of CyP functions by CsA may provoke pleiotropic downstream effects that may lead to the observed growth inhibition and loss of viability. In the context of the current literature, two pathways may be singled out with potential relevance for the CsA-dependent toxicity. First, *L. donovani* adenosine kinase aggregates have been identified as clients for CyP2, which disaggregates complexes of this protein [20], [66], thereby playing an important function in the purine salvage pathway [67]. Inhibition of this important CyP2 chaperone function may limit the intracellular concentration of adenosine and affect DNA synthesis with consequences for promastigote growth and amastigote viability. Second, cyclophilins have been reported to participate in the response to heat stress in other microbial pathogens. In the human pathogenic fungi *Cryptococcus neoformans*, CsA treatment prevents growth at elevated temperatures [68], [69] and the CyP-related protein Cp1a is required for full expression of fungal virulence [70]. Our data indeed established a direct link between the sensitivity of *Leishmania* to CsA and the parasite thermotolerance. We demonstrated that CsA-treated amastigotes are insensitive to the drug when incubated at 26°C, while CsA-resistant promastigotes are efficiently killed by the inhibitor at 37°C (Fig. 7). A second observation linked *Leishmania* CyPs with the response to increased temperature. We observed a striking effect of CsA on promastigote morphology, which acquired an oval cell shape and shortened their flagella, thus showing some (but not all) features characteristic for amastigote differentiation (Fig. 5). A similar morphogenic effect has been previously observed on promastigotes treated with the HSP90 inhibitor geldanamycin [53]. It is possible that both CsA and geldanamycin target different proteins are part of the same heat shock complex implicated in *Leishmania* differentiation and thermotolerance, such as cyclophilin 40, a multifunctional protein that interacts with various members of the HSP family through conserved TPR domains [58]. Indeed, our data identified LmaCyP40 as a direct target for CsA as judged from the direct interaction between the enzyme and the inhibitor (Fig. 8B) and CsA-dependent inhibition of LmaCyP40 PPIase activity (Fig. 8C). It is interesting to speculate that the temperature-dependent CsA effect on *Leishmania* viability is the result of CyP40 inhibition. Future studies employing LmaCyP40 conditional null mutants with the aim to dissociate the PPIase and chaperone functions of this enzyme may allow testing this hypothesis and shed important new light on the function of LmaCyP40 in parasite thermotolerance and infectivity.

In conclusion, our data revealed for the first time a direct cytostatic and cytotoxic effect of CsA on *L. donovani* in culture. We provided evidence that the stage-specific effects of CsA are governed by independent mechanisms linked to inhibition of calcineurin phosphatase activity in promastigotes, and inhibition of CyP functions relevant for thermotolerance in amastigote. We identified unique sequence elements in *Leishmania* CyPs and documented a considerable evolutionary expansion of this protein family, compared to other organisms, emphasizing the importance of this class of molecules for trypanosomatid-specific biology. The requirement of *Leishmania* CyP functions for intracellular parasite survival and their substantial divergence from host CyPs defines these proteins as prime drug targets. The suppressive action of CsA on host immunity and its exacerbating effects on murine toxoplasmosis, trypanosomiasis, and visceral leishmaniasis [24], [71], [72] obviously eliminates this drug for anti-parasitic intervention. Hence, the focus of future research should lie on the identification of novel CyP inhibitors that specifically target parasite CyPs without altering the host immune status.

## Supporting Information

Dataset S1 MALDI-ToF-ToF mass spectrometry analysis of promastigote lysate bound to CsA-coupled resin.
(0.04 MB XLS)

Figure S1 Recombinant GST::Strep::CyP40 was extracted from transformed *E. coli*, digested with factor Xa, purified by FPLC (A) as described in materials and methods, and fractions A7–A9 were pooled and analyzed by SDS-PAGE and coomassie staining (B). M, marker; F, pooled fractions.
(0.66 MB TIF)
