# Supplementary figures and images for: Cyclosporin A Treatment of Leishmania donovani Reveals Stage-Specific Functions of Cyclophilins in Parasite Proliferation and Viability
Source: PLoS Negl Trop Dis. 2010 Jun 29;4(6):e729. doi: 10.1371/journal.pntd.0000729 (PMC2894131; doi:10.1371/journal.pntd.0000729)

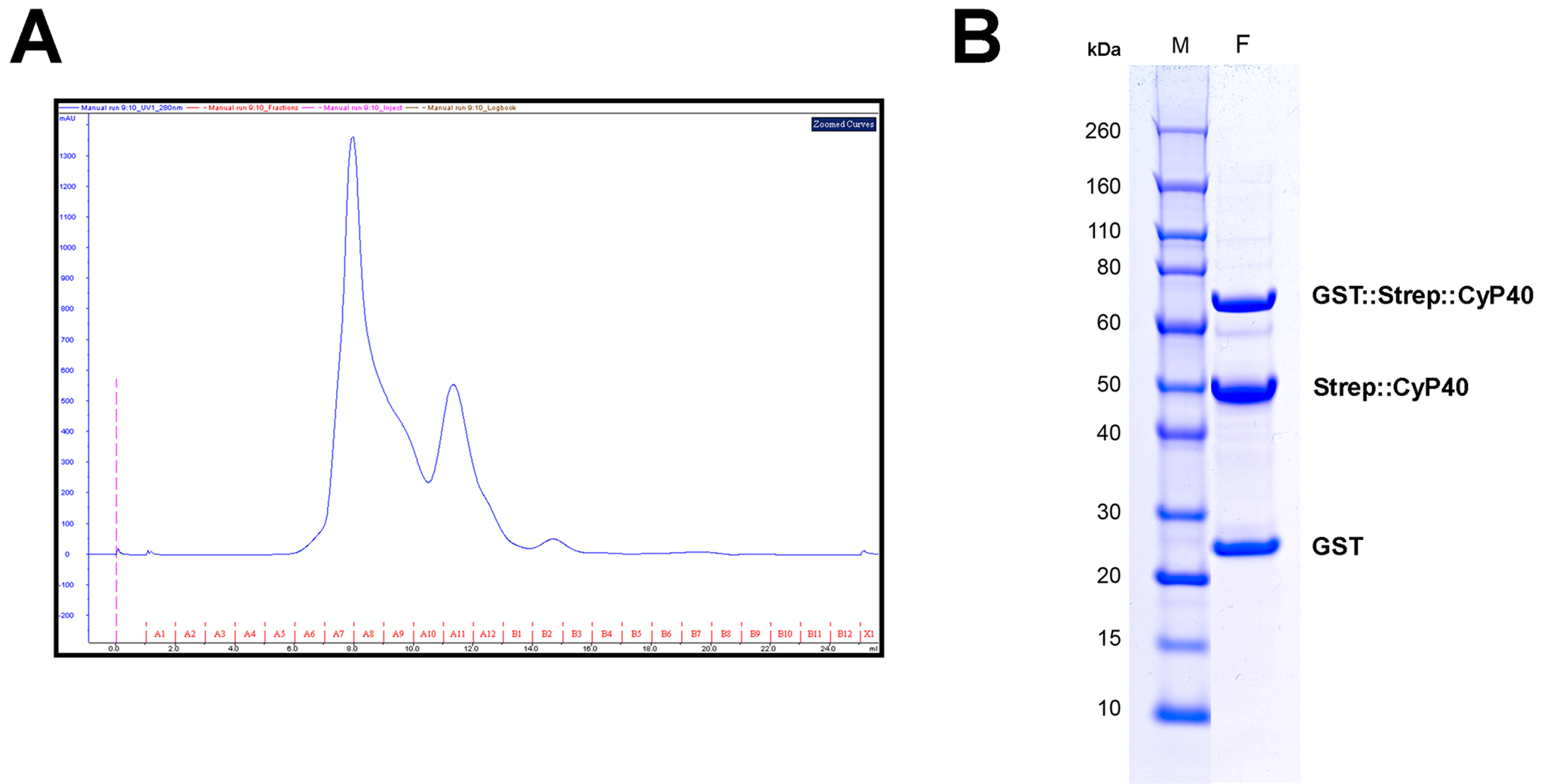

Supplement: Figure S1 — Recombinant GST::Strep::CyP40 was extracted from transformed E. coli, digested with factor Xa, purified by FPLC (A) as described in materials and methods, and fractions A7–A9 were pooled and analyzed by SDS-PAGE and coomassie staining (B). M, marker; F, pooled fractions. (0.66 MB TIF) [file pntd.0000729.s002.tif]
